# The treatment of behavioural and psychological symptoms in dementia: pragmatic recommendations

**DOI:** 10.1111/psyg.13116

**Published:** 2024-04-18

**Authors:** Camille Mercier, Victoria Rollason, Mohamed Eshmawey, Aline Mendes, Giovanni B. Frisoni

**Affiliations:** ^1^ Laboratory of Neuroimaging of Aging (LANVIE) University of Geneva Geneva Switzerland; ^2^ Memory Center, Department of Rehabilitation and Geriatrics University Hospitals of Geneva Geneva Switzerland; ^3^ Department of Acute Medicine, Clinical Pharmacology and Toxicology Service University Hospitals of Geneva Geneva Switzerland; ^4^ Department of Psychiatry, Geriatric Psychiatry Service University Hospitals of Geneva Geneva Switzerland; ^5^ Geriatrics and Rehabilitation Department, Department of Rehabilitation and Geriatrics University Hospitals of Geneva and University of Geneva Geneva Switzerland

**Keywords:** BPSD, dementia, non‐pharmacological interventions, pharmacological treatment, practice recommendations

## Abstract

Behavioural and psychological symptoms of dementia (BPSD) are a clinical challenge for the lack of a sound taxonomy, frequent presentation with comorbid BPSD, lack of specific pharmacologic interventions, poor base of methodologically sound evidence with randomized clinical trials, contamination from the treatment of behavioural disturbances of young and adult psychiatric conditions, and small efficacy window of psychotropic drugs. We present here a treatment workflow based on a concept‐driven literature review based on the notions that (i) the aetiology of BPSD can be mainly neurobiological (so‐called ‘primary’ symptoms) or mainly environmental and functional (‘secondary’ symptoms) and that this drives treatment; (ii) the clinical efficacy of psychotropic drugs is driven by their specific profile of receptor affinity; (iii) drug treatment should follow the rules of ‘start low–go slow, prescribe and revise’. This article argues in support of the distinction between primary and secondary BPSD, as well as their characteristics, which until now have been just sketchily described in the literature. It also offers comprehensive and pragmatic clinician‐oriented recommendations for the treatment of BPSD.

## INTRODUCTION AND AIM

Almost all patients with dementia develop behavioural and psychological symptoms of dementia (BPSD) during the course of their illness. These symptoms often result in major distress for the patient and their family, high care costs, and premature institutionalization. These symptoms are characterized by disturbances of perception (hallucinations), mood (depression, anxiety), thought content (delusions) and behaviour (disinhibition, agitation, aggression, apathy), or physiological functions (insomnia, appetite problems). However, BPSD affect over 90% of patients with dementia, appear at different stages and may be more or less predominant depending on the type of dementia.^1^, ^2^

In the case of Alzheimer's disease (AD), depression and anxiety are the most frequent symptoms in the early stages, making it difficult to establish a differential diagnosis between AD and psychopathological disorders.^2^, ^3^ These same symptoms then tend to worsen in the middle stages of AD and become progressively less manifest in the advanced stages, giving way to agitation, aggressivity, and so‐called ‘psychotic’ symptoms (hallucinations and delusions). In dementia as a whole, agitation and irritability are the most common symptoms, affecting more than one in two patients.^1^ Significant persistence and incidence are associated with apathy, while also present at a low to moderate degree for affective and psychotic symptoms.^4^ In addition, some symptoms such as hallucinations may disappear spontaneously, underlining the importance of systematically considering withdrawal of psychotropic drugs at some point during medical monitoring in the case of management including pharmacological treatment.^5^

Several guidelines and recommendations have been developed to help clinicians identify the most appropriate intervention. Virtually all stipulate that non‐pharmacological interventions (reduction of sensory input and cognitive stress, sensory stimulation interventions, physical exercise, reminiscence therapy, animal‐assisted therapy) should be first line and pharmacological interventions (neuroleptics, SSRIs, benzodiazepines, hypnotics, etc.) second line to minimize the impact of adverse effects of the latter. However, the observation of daily practice indicates that most patients receive inappropriate or downright harmful pharmacologic interventions.^6^ While a number of clinical, scientific, and organizational factors contribute to this unwelcome outcome, we believe that insufficient clarity on the taxonomy and pathophysiology of BPSD contributes at least in part to it.

The aim of this paper is to propose a novel taxonomy of BPSD where these are categorized into primary and secondary, where the former have a mainly neurobiological aetiology and first‐line interventions are pharmacological, and the latter are mainly reactive to external stressors or functional impairment and first‐line interventions are mainly environmental. We further offer an evidence‐based approach to drug choice based on neurochemical affinity and pharmacodynamics profile, assuming that BPSD have a pathophysiology distinct from that of psychiatric disturbances bearing the same name, and relying on a principle of clinical economy that minimizes polypharmacy.

## PATHOPHYSIOLOGY

Despite sharing the same name of ‘psychotic symptoms’, these disorders in dementia and psychiatric pathologies (schizophrenia, bipolar disorders) differ in terms of their pathophysiology, neurological involvement and consequently, their treatment. These two groups do not present the same profile of psychotic symptoms, which are usually both less clinically complex and less organized in patients suffering from a dementia pathology than in non‐demented patients. While the usual psychotic manifestations of schizophrenia include the perception of one or more voices, this phenomenon is only rarely observed in dementia^7^ whose symptoms are difficult to differentiate from confabulations (spontaneous production of stories to fill in gaps in autobiographical memory),^8^ particularly in the more advanced stages of cognitive impairment. The psychotic symptoms characteristic of dementias commonly take the form of delusions of theft, infidelity, or misidentification of people.^7^, ^8^, ^9^ Hallucinations, when present, are generally visual and occur in Lewy body dementia (LBD).^10^

BPSD can be divided into two categories, primary and secondary, which differ both in their origin and in the way they are treated. Primary BPSD result from the process of neurodegeneration affecting neuronal networks and neurotransmission systems and mainly concern frontotemporal dementia (FTD) and LBD, whereas secondary BPSD, which predominate in the early to intermediate stages of AD, result from a psychological reaction to cognitive decline.^3^, ^11^, ^12^ However, as AD progresses, the development of BPSD gradually tends to be underpinned by biological causes.^3^ Primary BPSD occur spontaneously, in contrast to secondary BPSD, which are significantly underpinned by both disease‐induced functional impairment and the Progressively Lowered Stress Threshold (PLST) model. According to this theory, patients affected by dementia progressively lose the ability to receive, process, and respond to environmental stimuli.^13^ These difficulties stem directly from the cognitive loss that accompanies the disease and result in the emergence of dysfunctional behaviours. In this context, stressors can take many different forms and arise from internal demands (e.g. fatigue, illness, hunger, reactions to medication) or external demands (e.g. noise, change of carer or routine) that exceed the patient's ability to adapt.^13^ The nature of some BPSD is twofold and can be both primary and secondary, depending on the dementia and the stage at which they appear in the course of the disease. The origins of the genesis of BPSD in AD (early to intermediate stages), FTD, and LBD are developed in the supplementary material section Data **S1**, and figures relating to prevalence, incidence, and persistence are reported for the main symptoms in Table 1.^1^, ^4^ In fact, assessing the nature of the BPSD expressed by the patient is the first step in selecting the most appropriate treatment (pharmacological or non‐pharmacological), the full procedure for which is described in Table 2.

**Table 1:** Prevalence, incidence, and persistence of behavioural and psychological symptoms of dementia (BPSD) by type of dementia^1^, ^4^

Nature of BPSD	Alzheimer disease	Frontotemporal dementia	Dementia with Lewy bodies	All dementias combined	
	Prevalence	Prevalence	Prevalence	Incidence (over 3 months or more)	Persistence (over 3 months or more)
Delusions	35%	20%	49%	Moderate (5–84%)	Low (0–82%)
Hallucinations	24%	13%	61%	Low (4–45%)	Low (0–52%)
Agitation	61%	63%	56%	High (19–80%)	Moderate (21–77%)
Depression	51%	40%	57%	Moderate (10–73%)	High (16–70%)
Anxiety	46%	39%	41%	Moderate (12–38%)	High (17–52%)
Sleep disorders	36%	36%	40%	Low (8–31%)	Low (10–57%)

**Table 2:** Steps to select the most appropriate drug(s) to treat behavioural and psychological symptoms of dementia (BPSD) in individual patients

Inventory of patient's BPSD	1. Are BPSD primary or secondary?	2. Are drugs necessary?	3. Select a list of candidate drugs	4. Reduce number of drugs to a minimum (ideally one drug only)	Start treatment
	Table 3		Tables 4, 5, and 6		

Although they may be expressed via other sensory modalities, visual hallucinations are a frequent symptom of LBD from the prodromal stage and may be due to an alteration in the cholinergic/dopaminergic balance, to alpha‐synuclein deposits in the brain regions responsible for perception, such as the visual cortex, and to impairment of neuronal communication between the neocortex and the limbic system (amygdala and hippocampus).^10^ Delusions affect around 33% of patients with LBD including misidentifications and delusions of infidelity, although the most common are delusions of theft, persecution, and jealousy.^14^, ^15^ Misidentifications may take different forms such as the belief that a well‐known person has been replaced by a look‐alike (Capgras syndrome), the conviction that one is in a copy of a familiar place (reduplication of place), the inability to distinguish perceptually between fiction and reality (television sign) or to recognize one's own image in the mirror (the mirror sign). These symptoms may occur in parallel with the hallucinations, but they are also expressed individually and chronically in the course of LBD. The development of delusions in LBD may be underpinned by dysfunction of the right prefrontal cortex and temporal lobe, as well as hyperactivation of M1 muscarinic receptors.^14^, ^15^

Although psychotic symptoms are rarer in FTD, patients with the C9orf72 gene expansion are more likely to experience delusions, with a prevalence of 57% of carriers compared with 19% of non‐carriers.

C9orf72 expansion may cause altered body schema due to thalamo‐cortico‐cerebellar networks dysfunction, resulting in the development of delusions.^16^ Disinhibition, apathy, and agitation are major features of FTD and are often present from the earliest stages of the disease. They result from atrophy and neuronal loss in the frontal and temporal lobe networks involved in behavioural control, aggregates of abnormal tau or TDP‐43 proteins in these same regions, and serotonin and dopaminergic dysregulation involved in mood, impulsivity, and reward processing.^11^, ^12^ The same pathophysiology may be responsible for the hallucinations and disinhibition in some cases of atypical AD.^17^

However, the majority of psychotic symptoms observed in AD patients are secondary or reactive to the cognitive deficit.^3^, ^9^, ^18^ As a result of the memory loss induced by dementia, a patient may actually believe that a family member has stolen their belongings because they cannot remember where they were, or that a close relative has been replaced by a lookalike because they can no longer feel their emotional correlates.^13^, ^18^ Some studies have shown that delusions of theft, which are the most common in AD with a prevalence of 28%,^19^ are directly correlated with scores on tasks requiring episodic memory.^9^ Moreover, these findings are consistent with neuroimaging data showing hypoperfusion in the right medial posterior parietal cortex (a region known to be involved in episodic memory processing) in patients with delusions of theft.^9^ Greater cortical loss in the hippocampal region (heavily involved in episodic memory) was also found in AD patients suffering from misidentifications, highlighting the link between cognitive decline and the onset of delusions in patients with AD.^8^ Delusions of persecution affect around one in five of AD patients and constitute a specific primary symptom. They appear more spontaneously, earlier, and are not associated with a decline in episodic memory but caused by frontal atrophy and dysregulation of the cortical and subcortical serotonin system.^8^ In the early to intermediate stages of AD, symptoms of anxiety or agitation often accompany and are therefore secondary to delusions.^18^, ^20^ On the contrary, in FTD or LBD, these same manifestations may directly result from biological damage. In FTD, agitation might be directly associated with dysregulation of the dopaminergic and serotonergic neurotransmission systems,^11^ whereas in LBD, this symptom may result from dysfunction of the temporal lobe and fusiform gyrus caused by the local propagation of Lewy body pathology.^21^

Affective disorders often appear early in AD and LBD but differ in severity and phenotype during the initial phase of dementia progression. In AD, depressive symptoms are of moderate severity at the onset of the disease and result mainly from difficulties in adapting to cognitive loss.^3^ Horning *et al*. demonstrated an association between patient awareness and the risk of incident anxiety and depressive symptoms.^22^ The results of Zahodne *et al*.'s study are in line with previous findings, showing that depression may not only be an affective reaction to memory problems, but may also be attributable to the patient's loss of functional abilities.^23^ Moreover, early stages of AD seem to be characterized by anxious manifestations that may result from initial compensatory behaviour and loss of self‐sufficiency.^3^ Indeed, some AD patients experience an increase in anxiety after being separated from their carer.^20^ In LBD, affective disorders are more severe and persistent than in other dementias. Depressive symptoms, present in more than 50% of LBD patients, may be generated by the combination of serotonin dysfunction associated with the accumulation of Lewy bodies in the Raphe nucleus and the reduction in dopaminergic transmission characteristic of this disease.^15^, ^24^ Anxiety often accompanies visual hallucinations, sometimes in acute forms such as panic attacks, but can also be expressed in a chronic and more discreet way (e.g. numerous demands on loved ones, feeling of abandonment).^15^ Moreover, anxiety in LBD might result from early pathogenic mechanisms in the cortico‐limbic system and the corresponding neurotransmitter systems.^25^ Although generally less frequent in FTD than in other dementias, affective symptoms may be explained by a disturbance in the serotonin system which is significantly more affected than in other dementias such as AD.^26^

Rapid eye movement (REM) sleep disorders affect approximately 40% of LBD patients who express characteristic symptoms such as dreamlike agitation, somniloquy, or screaming caused by the loss of skeletal muscle atonia normally present. They are due to degeneration of the brainstem and its impact on various structures involved in muscle atonia, such as the pedunculopontine nucleus, the magnocellular reticular formation, and the sublaterodorsal nucleus.^15^, ^27^ Around 35% of FTD patients present mild and clinically significant sleep disorders^1^ which are present throughout the course of the disease including fragmented sleep and excessive daytime sleepiness. They may be explained by a disturbance in orexin, a hypothalamic hormone directly involved in sleep control. In fact, FTD is associated with a reduction in the posterior part of the hypothalamus.^28^ Sleep disorders are predominantly primary in AD, appearing only from a moderate stage of severity and then gradually worsening as the disease progresses.^29^

While priority is given to psychoeducation and, more generally, to all non‐pharmacological alternatives in the management of secondary BPSD, the drug approach is often introduced early in the case of primary BPSD in combination, wherever possible, with non‐pharmacological measures. Furthermore, premorbid personality influences the phenotype of secondary BPSD by shaping the individual's responses to the cognitive decline. Neuroticism has been shown to be the most influential personality factor in the development of affective disorders (in particular depression) and behavioural symptoms in AD, which may be mediated by greater emotional sensitivity and poorer stress management. On the other hand, AD patients with a higher level of premorbid conscientiousness may develop less BPSD overall due to better self‐control (Table 3).^30^

**Table 3:** Characteristics of primary and secondary behavioural and psychological symptoms of dementia (BPSD)^1^, ^2^, ^3^, ^4^, ^5^, ^6^, ^7^, ^8^, ^9^, ^10^, ^11^, ^12^, ^13^, ^14^, ^15^, ^16^, ^17^, ^18^, ^19^, ^20^, ^21^, ^22^, ^23^, ^24^, ^25^, ^26^, ^27^, ^28^, ^29^, ^30^, ^31^, ^32^, ^33^

BPSD	Primary	Secondary
Clinical epidemiology^2^, ^3^	Less common	Frequent
Taxonomy^2^, ^3^, ^7^, ^8^, ^9^, ^10^, ^11^, ^12^	Lewy body disease, Frontotemporal degeneration	Alzheimer's disease (early stage)
Pathophysiology^8^, ^9^, ^10^, ^11^, ^12^, ^13^, ^14^, ^15^, ^16^, ^17^, ^18^, ^19^, ^20^, ^21^, ^22^, ^23^, ^24^, ^25^, ^26^, ^27^, ^28^, ^29^	Biological damage (dorsolateral prefrontal cortex, orbital cortex, anterior cingulate cortex, temporal poles)	Psychological reaction
Contribution of pre‐morbid personality^30^	Not significant	Significant
Phenotype^2^, ^3^, ^8^, ^9^, ^10^, ^11^, ^12^, ^13^, ^14^, ^15^, ^16^, ^17^, ^18^, ^19^, ^20^, ^21^, ^22^, ^23^, ^24^, ^25^, ^26^, ^27^, ^28^, ^29^	Disinhibition, Hallucinations, Physical aggression, Insomnia, Apathy	Depression, Apathy, Denial, Aggression, Dependence on caregivers, Delusions (theft, jealousy)
Onset^3^, ^8^, ^9^, ^10^, ^11^, ^12^, ^13^, ^14^, ^15^, ^16^, ^17^, ^18^, ^19^, ^20^, ^21^, ^22^, ^23^, ^24^, ^25^, ^26^, ^27^, ^28^, ^29^	Spontaneous	Functional impairment, Environmental stress
Severity^32^, ^33^	All levels of severity	Mild to moderate
Clinical observation^8^, ^9^, ^10^, ^11^, ^12^, ^13^, ^14^, ^15^, ^16^, ^17^, ^18^, ^19^, ^20^, ^21^, ^22^, ^23^, ^24^, ^25^, ^26^, ^27^, ^28^, ^29^, ^32^, ^33^	Socially disturbed	Socially appropriate
Social competence^31^	Impairment	Preserved
First‐line treatment^34^, ^35^, ^36^, ^37^, ^38^, ^39^, ^40^, ^41^, ^42^, ^43^, ^44^, ^45^, ^46^, ^47^, ^48^, ^49^, ^50^	Medication	Psychoeducation
Second‐line treatment^34^, ^35^, ^36^, ^37^, ^38^, ^39^, ^40^, ^41^, ^42^, ^43^, ^44^, ^45^, ^46^, ^47^, ^48^, ^49^, ^50^	Psychoeducation	Medication

## TREATMENT: BASIC RECOMMENDATIONS

In principle, the non‐pharmacological approach should always be considered as the first line in the management of BPSD. However, there are a number of situations that justify the early use of drug options, whose introduction may be justified in synergy with that of non‐pharmacological interventions.^34^, ^35^, ^36^, ^37^, ^38^, ^39^, ^40^, ^41^, ^42^, ^43^, ^44^, ^45^, ^46^, ^47^, ^48^, ^49^, ^50^

When faced with a patient presenting with BPSD, the first step in the clinical approach is to assess the underlying causes in order to rule out any iatrogenic, somatic, or environmental origin, unless the patient manifests an imminent danger to themselves or others, in which case they should be treated immediately.^34^, ^35^ Indeed, untreated pain is the most frequent cause of BPSD, and must therefore be systematically assessed before any other treatment is initiated. Other factors such as infections or the adverse effects of pharmacological treatments often produced by benzodiazepines, hypnotics, tricyclic antidepressants, and others can also be the cause of BPSD.^34^, ^35^, ^40^ In this case, the chronological link between the start of treatment or dose increase and BPSD will be crucial to assess.

## NON‐PHARMACOLOGICAL TREATMENT

Among the most common underlying causes of BPSD are environmental stimuli that are incongruous or excessive for the patient. These include noises that are too loud (e.g. several people talking out loud), excessive heat, scenes that are incomprehensible to the patient, violent or disturbing (e.g. a war film on TV, their own image in a mirror), reproaches or requests from the carers that the patient is unable to understand (e.g. taking a shower or getting dressed for bed whereas the patient has lost track of time) are all potential causes of agitation or anxiety.^36^, ^40^ It is clear that the treatment of BPSD associated with environmental stimulation involves eliminating the cause, which is often done through psycho‐education of the family carer.^34^

Most recommendations from scientific societies and expert groups agree that the degree of severity of BPSD and patient distress should guide treatment, with severe BPSD requiring priority treatment with psychotropic drugs, while mild to moderate BPSD should be treated primarily with non‐pharmacological interventions. Details of these recommendations from leading expert societies can be found in the supplementary material section Data S1.^37^, ^38^, ^39^, ^40^, ^41^, ^42^, ^43^, ^44^, ^45^, ^46^, ^47^, ^48^, ^49^, ^50^ However, in most cases, the manifestations of secondary BPSD are mild to moderate, unlike primary BPSD, which can be of any level of severity.^32^, ^33^ At present, various non‐pharmacological practices have proved their worth in the management of BPSD, falling into different categories according to their approach.^132^

There are a variety of behavioural interventions for people with dementia, such as problem‐solving techniques, social skills training, token‐saving systems, occupational therapy, and cognitive behavioural therapy.^132^ In a controlled clinical study, both patients and family carers who had undergone behavioural treatments involving the programming of pleasurable activities and problem‐solving techniques showed a reduction in their depressive symptoms after 6 months.^133^ An increasing body of scientific evidence now supports the efficacy and central role of family therapy and psycho‐education of family carers in the patient's therapeutic process.^34^ For example, some studies have shown that taking care of the family can both postpone institutionalization and promote successful integration within the institution if it is introduced.^134^, ^135^

Emotionally‐oriented approaches include a number of techniques such as reminiscence therapy and the validation method, which have been shown to reduce agitation and depression in dementia patients. Reminiscence therapy involves discussing past experiences using a variety of media, such as photographs or familiar objects, which encourage the patient to generate memories. Validation therapy aims to facilitate the expression and acceptance of dementia patients' feelings by adopting a gentle attitude and voice and using simple, concrete words.^132^

Many approaches based on sensory stimulation, such as aromatherapy, massage, light therapy, dance, music, and gardening have been shown to have beneficial effects on certain BPSD including anxiety, depression, and agitation. Multisensory stimulation therapy, also known as ‘Snoezelen’, falls into the same category and aims to gently stimulate all the patient's sensory modalities using a specific framework. In fact, this procedure takes place in a room offering the patient a calm atmosphere and a variety of sensory aids such as aromas, music, bubble tubes, or even drawings projected onto the walls.^132^

Other non‐specific interventions such as animal‐assisted therapy, doll therapy, and physical exercise have shown benefits for some BPSD.^136^, ^137^, ^138^, ^139^ In a randomized controlled trial (RCT), 30 patients with dementia received several 45‐min sessions once a week for 10 weeks during which they were invited to interact with a dog by petting it or throwing balls for it. In the control group, symptoms of agitation and depression worsened over the course of the study, unlike the patients who received the intervention, for whom these disorders remained stable.^136^ These results suggest that animal‐assisted therapy could slow the progression of some BPSD and thus constitute an interesting non‐pharmacological option. Although this method is subject to a great deal of controversy, studies show that doll therapy is useful in cases of agitation, anxiety, dysphoria, or apathy in patients with moderate to severe dementia. The benefits of this intervention are twofold: It focuses the patient's attention on a soothing stimulus and, at the same time, gives them a feeling of usefulness.^137^, ^138^

## PHARMACOLOGICAL TREATMENT

If the patient expresses significant distress or severe symptoms, which is often the case in primary BPSD, pharmacological treatment with psychotropic drugs is recommended. Drug treatment(s) should be initiated at low doses and increased gradually (‘start low, go slow’).^40^, ^43^ The tolerability of the psychotropic drugs introduced and their efficacy in relation to the precise objective defined in agreement with the patient and the family should also be assiduously assessed, at least every 3 months (‘prescribe and revise’).^40^, ^49^ All of the patient's symptoms must be taken into account to guide the choice of the most appropriate drug treatment(s) because some medications have secondary indications that enable them to act on several symptoms at once. In particular, when certain symptoms are associated with depression, such as agitation or anxiety, these may also respond favourably to certain classes of antidepressants, such as trazodone or mirtazapine (‘two birds with one stone’).^36^, ^49^

The various recommendations for good practice relating to the use of psychotropic drugs in general, and antipsychotics in particular, are convergent and give precise instructions. If no clinical improvement is observed 4 weeks after initiation of antipsychotic treatment at an adequate dose, weaning from the drug should be considered.^38^, ^48^ It is also strongly contraindicated to add a second psychotropic drug if the first has not shown any beneficial effect, but rather to introduce another molecule gradually as the first pharmacological treatment is reduced.^44^, ^49^ Because of the adverse effects associated with antipsychotic treatment (e.g. sedation, increased risk of falls, extrapyramidal effects, accelerated cognitive decline, stroke, increased mortality), antipsychotic treatment should be systematically withdrawn within 4 months after initiation^38^ unless the patient has experienced a recurrence of symptoms during two previous attempts at withdrawal and non‐pharmacological interventions and alternative psychotropic medications have proved ineffective.^38^, ^50^ In addition, treatment tapering should be slow in order to monitor for any relapse of symptoms, unless the patient is experiencing severe antipsychotic‐induced adverse effects.^50^ Any attempt to reduce psychotropic treatment should be carried out with the agreement of family carers and the patient whenever possible.^37^, ^50^

Almost all psychotropic drugs have a multi‐transmitter/receptor mechanism of action because of their relatively low selective affinity for stimulating or blocking different neurotransmitter/receptor systems (cholinergic, serotonergic, dopaminergic, etc.). Rational pharmacological treatment of BPSD must be developed on the basis of sound knowledge of the clinical effects of stimulating and blocking neurotransmitter systems and of the affinity of each molecule for stimulating and blocking each neurotransmitter system. Table 4 summarizes the effects of stimulating and blocking the cholinergic, serotonergic, dopaminergic, GABAergic, and histaminergic systems, while Table 5 describes the affinity of the most commonly used classes of psychotropic drugs for the neurotransmitter systems. The two tables provide a guide to the clinical effects of the classes of psychotropic drugs most commonly used in clinical practice (Table 6). It should be noted that knowledge of the affinity of psychotropic drugs is imperfect, as studies are generally carried out in animals, whereas clinical studies are still the best way of verifying the accuracy of indications. In Table 5, we have tried to balance the empirical evidence with the effects actually observed in humans.

**Table 4:** Neurotransmitter systems: stimulation and blockade

Neurotransmitter	Stimulation	Blockade
Acetylcholine^51^, ^52^	Bradycardia, increased sweating and salivation, diarrhoea, miosis	Anticholinergic syndrome, either: Confusion, dry mouth, mydriasis, blurred vision, increased heart rate, urinary retention, constipation, increased intraocular pressure (glaucoma)
Serotonin^53^	Serotonin syndrome, either: Elevated body temperature, agitation, increased reflexes, tremor, sweating, dilated pupils, diarrhoea	‐
Dopamine^51^, ^54^, ^55^, ^56^	Hallucinations, agitation, euphoria, pathological gambling	Parkinsonism (rigidity, akinesia, stooped posture, dysarthria etc.)
Noradrenaline^51^, ^57^, ^58^	Tachycardia, sweating, palpitations, anxiety, headaches, low blood sugar, loss of appetite	Decreased heart rate, hypotension, depression
GABA^51^	‐	Decreased anxiety, sleep, falls
Histamine^51^, ^59^	‐	Sedation, confusion

**Table 5:** Effects of psychotropic drugs on neurotransmission systems

	ACh	Serotonin	DA	NA	GABA	Histamine
Anxiolytics (BZD)^51^	No	No	No	No	+++	No
Hypnotics (Z‐drugs)^51^, ^60^	No	No	No	No	+++	No
Ant. Tricyclics^61^, ^62^, ^63^, ^64^, ^65^, ^66^	‐‐	+/+++	+	+/+++	No	‐‐‐
SSRIs (citalopram, escitalopram, sertraline)^61^, ^62^, ^63^, ^64^, ^65^, ^66^, ^67^, ^68^, ^69^, ^70^, ^71^, ^72^, ^73^, ^74^	No (except paroxetine)	+++	‐	No/+	No	No/‐
SNRIs (venlafaxine, milnacipran)^61^, ^62^, ^63^, ^73^, ^74^, ^75^	No	+++	+/‐	+++	No	No
Trazodone^61^, ^62^, ^63^, ^76^	No	+/‐ †	‐‐	No	No	‐‐
Mirtazapine^61^, ^62^, ^63^, ^77^	No	+++	No	+++	No	‐‐‐
Haloperidol^78^	No	‐	‐‐‐	No	No	‐
Risperidone^78^, ^79^	No	‐‐‐	‐‐‐	No	No	‐‐‐
Quetiapine^78^	‐	‐‐	‐	‐	No	‐‐‐
Olanzapine^78^	‐‐	‐‐‐	‐‐‐	‐	No	‐‐‐
Clozapine^78^	‐‐‐	‐‐‐	‐	‐	No	‐‐‐
Antihistamines^51^, ^80^	‐‐	No	No	No	No	‐‐‐

*Note*: No: no effect on neurotransmitter action; increased neurotransmitter action (+ weakly; ++ moderately; +++ strongly); decreased neurotransmitter action (‐ weakly; ‐‐ moderately; ‐‐‐ strongly); ‐/no: no effect or slightly decreased neurotransmitter action; no/+: no effect or slightly increased neurotransmitter action. No/+: no effect or slightly increased action of neurotransmitter.

Abbreviations: Ach, acetylcholine; Ant, antidepressant; BZD, benzodiazepine; DA, dopamine; SNRIs, serotonin‐noradrenaline reuptake inhibitors; NA, noradrenaline; SSRIs, selective serotonin reuptake inhibitors.

^†^ +/‐ action of neurotransmitter decreased or increased depending on dose.

**Table 6:** Beneficial and undesirable clinical effects of psychotropic drugs in humans

Drug	Delusions/Hallucinations	Anxiety	Depression	Sleepiness	Agitation	Parkinsonism	Weight gain	Orthostatic hypotension	QT interval prolongation	Other
Anxiolytics (BDZ)^81^, ^82^, ^83^, ^84^, ^85^, ^86^	May be detrimental	+++	May be detrimental in the long term	+++	May be detrimental	No effects	No effects	No effects	No effects	Addiction; paradoxical reactions; risk of falls
Hypnotics (non BDZ)^81^, ^85^, ^86^, ^87^, ^88^, ^89^, ^90^	May be detrimental	No effects	No effects	+++	May be detrimental	No effects	No effects	No effects	No effects	Addiction; paradoxical reactions; risk of falls
Tricyclic antidepressants^85^, ^86^, ^91^, ^92^, ^93^, ^94^, ^95^	May be detrimental	+	+++	++	May be detrimental	No/+	+/++	+/+++	+/++	High risk of acute confusional state, contraindicated
SSRIs^85^, ^86^, ^91^, ^92^, ^94^, ^95^, ^96^, ^97^, ^98^, ^99^, ^100^	Not known	++	+++	+/‐	++	No/+	+/‐	No	+/+++	Hyponatremia
SNRIs ^64^, ^65^, ^85^, ^86^, ^94^, ^95^, ^101^, ^102^	Not known	+	+++	+/‐	‐‐	No/+	No effects	No/+	No/++	Hypertension
Trazodone ^85^, ^86^, ^94^, ^95^, ^103^, ^104^, ^105^, ^106^	No effects	+++	+	+++	++	No/+	+/‐	++	+	Priapism
Mirtazapine^61^, ^85^, ^86^, ^94^, ^95^, ^106^	No effects	+++	+++	+++	+	No/+	++	++	++	
Haloperidol^85^, ^86^, ^94^, ^107^, ^108^, ^109^	++	+	No effects	‐	++	+++	++	No effects	+++	
Risperidone^85^, ^86^, ^94^, ^109^, ^110^, ^111^, ^112^, ^113^, ^114^	+++	+	+	‐	+++	++	++	+	+	
Quetiapine^85^, ^86^, ^94^, ^108^, ^109^, ^115^, ^116^	+	+++	+	+++	+++	+	++	++	+	
Olanzapine^85^, ^86^, ^94^, ^109^, ^117^, ^118^, ^119^, ^120^	++	+	+	+	++	+	+++	+	+	
Clozapine^85^, ^86^, ^94^, ^109^, ^120^, ^121^, ^122^	++	+	+	+	++	No/+	++	+	++	Risk of agranulocytosis
Antihistamines^84^, ^85^, ^86^, ^94^, ^123^, ^151^	No effects	++	No effects	++	+	No effects	+	+	+	Risk of falls
Anticholinesterasiques^85^, ^94^, ^124^, ^125^, ^126^, ^127^, ^128^	++	+	+	May be detrimental	May be detrimental	No/+	‐	No effects	+/+++	Vomiting; Risk of falls
Memantine^85^, ^129^, ^130^, ^131^	+	No effects	No effects	+	+	No effects	No effects	No effects	No effects	

*Note*: + mild effect; ++ moderate effect; +++ significant effect; ‐ mild inverse effect; ‐‐ moderate inverse effect; ‐‐‐ significant inverse effect; no/+ absence of mild effect; no/++ absence of moderate effect; no/+++ absence of significant effect; +/‐ possible clinical effect or inverse clinical effect.

Abbreviations: BZD, benzodiazepine; SNRIs, serotonin‐norepinephrine reuptake inhibitors; SSRIs, selective serotonin reuptake inhibitors.

Selective serotonin reuptake inhibitors (SSRIs) are still the first choice for the treatment of depression since they are associated with fewer adverse effects than other classes of antidepressants such as tricyclics and present less risk of drug interactions.^91^, ^92^, ^96^, ^97^, ^98^, ^99^ In addition, some studies show beneficial effects of SSRIs on psychotic symptoms and agitation, particularly for citalopram.^100^ Trazodone is widely used for agitation, insomnia, and anxiety because of its sedative properties and negligible anticholinergic risk.^103^, ^104^, ^105^, ^106^ On the other hand, if the patient suffers from depression accompanied by other specific BPSD, alternatives to SSRIs may be considered. Trazodone and mirtazapine are recommended when agitation or anxiety accompany depression, with trazodone being particularly recommended for depression associated with agitation.^36^, ^49^, ^103^, ^104^, ^105^, ^106^, ^140^

Although many are not approved for use in elderly patients with dementia in many countries, such as Switzerland, atypical antipsychotics have been shown to be effective in reducing psychotic symptoms and agitation.^110^, ^111^, ^112^, ^113^, ^115^, ^117^, ^118^, ^119^, ^120^, ^121^, ^122^ However, the risk–benefit ratio needs to be carefully assessed because of the various dangers to which they may expose patients, such as increased mortality and risk of stroke, or accelerated cognitive decline.^141^ Risperidone is the atypical antipsychotic with the most published evidence in the literature, and its effects are supported by numerous RCTs (although the effect in RCTs is rather weak).^35^, ^142^ Although sometimes divergent, the studies tend to show a superiority of risperidone and olanzapine in reducing psychotic symptoms compared with quetiapine.^142^ Risperidone and olanzapine cause extrapyramidal symptoms more frequently than quetiapine, and olanzapine is the antipsychotic most likely to lead to weight gain.^142^ Given the high sensitivity of patients with LBD or Parkinson's disease dementia to antipsychotics, anticholinesterase agents such as rivastigmine or donepezil should be offered as first‐line treatment for delirium, hallucinations, and agitation.^40^, ^49^ If the use of anticholinesterase agents proves insufficient, clozapine or quetiapine are the only antipsychotics that can be prescribed due to their low risk of aggravating the extrapyramidal symptoms inherent in these two pathologies.^40^, ^49^ Although some studies suggest a positive response of anxiety to antipsychotic treatment,^36^ expert guidelines advise against long‐term use of this class of drugs.^38^, ^50^ Several studies have also demonstrated the efficacy of memantine in treating hallucinations, delusions, and agitation.^129^, ^130^

On the other hand, many good practice recommendations contraindicate the use of certain treatments in the management of BPSD. Certain classes of hypnotics, such as benzodiazepines and Z‐drugs, are not recommended, as they carry risks of addiction, confusion, paradoxical reactions, and falls, to which the elderly are more prone than the rest of the population.^36^, ^49^, ^81^ While anxiety or sleep disorders respond favourably to the latter drugs,^82^ their use should only be considered in exceptional circumstances (e.g. hospitalization) and on a short‐term basis.^49^ Similarly, tricyclic antidepressants, paroxetine, and antihistamines are also contraindicated in the elderly because of their anticholinergic properties, which often lead to acute confusional states (delirium).^34^, ^40^ Compared with atypical antipsychotics, haloperidol is more likely to produce extrapyramidal symptoms, and should therefore be avoided in the pharmacological management of non‐emergency BPSD.^36^, ^38^, ^40^, ^48^

## FUTURE PROSPECTS AND CONCLUSIONS

There is still considerable room for improvement in the treatments currently available, and further research in this area is essential in view of the increase in the number of cases of dementia due to the ageing of the population. At the same time, certain compounds could prove promising for different types of BPSD.

Suvorexant, an orexin receptor antagonist, could be a promising molecule for managing sleep disorders in Alzheimer's. A 4‐week clinical trial evaluating the effect of this drug by polysomnography in patients meeting the diagnostic criteria for insomnia and probable Alzheimer's dementia demonstrated an increase in total sleep time.^143^ Several studies aimed at measuring the efficacy of certain psychostimulants on apathy, such as methylphenidate, have shown beneficial effects in patients affected by AD.^144^, ^145^ Other research has also been carried out with modafinil, but the results obtained were not as conclusive.^144^

Certain antipsychotics could also be potential pharmacological treatments for the management of certain BPSD. The results of two phase 3 clinical trials conducted in parallel over a period of 12 weeks demonstrated the good tolerability and efficacy of the new antipsychotic brexpiprazole for agitation in AD.^146^, ^147^ Pimavanserin has also received considerable attention following its recent approval in the US for the treatment of psychotic symptoms associated with Parkinson's disease and its potential clinical value in treating the same symptoms in AD.^34^, ^147^, ^148^, ^149^ However, a clinical trial of pimavanserin was conducted for psychotic symptoms in AD, but the results were mixed, with positive effects observed after 6 weeks but not persistent after 12 weeks.^148^ Finally, in a preliminary phase 2 clinical trial, the administration of dextromethorphan‐quinidine to patients with AD over a period of 10 weeks showed a significant reduction in the level of agitation and relatively good tolerance.^150^

It should be nevertheless stressed that more empirical evidence is needed before these new drugs can be incorporated into the clinical algorithm for the treatment of BPSD.

**Figure 1:**
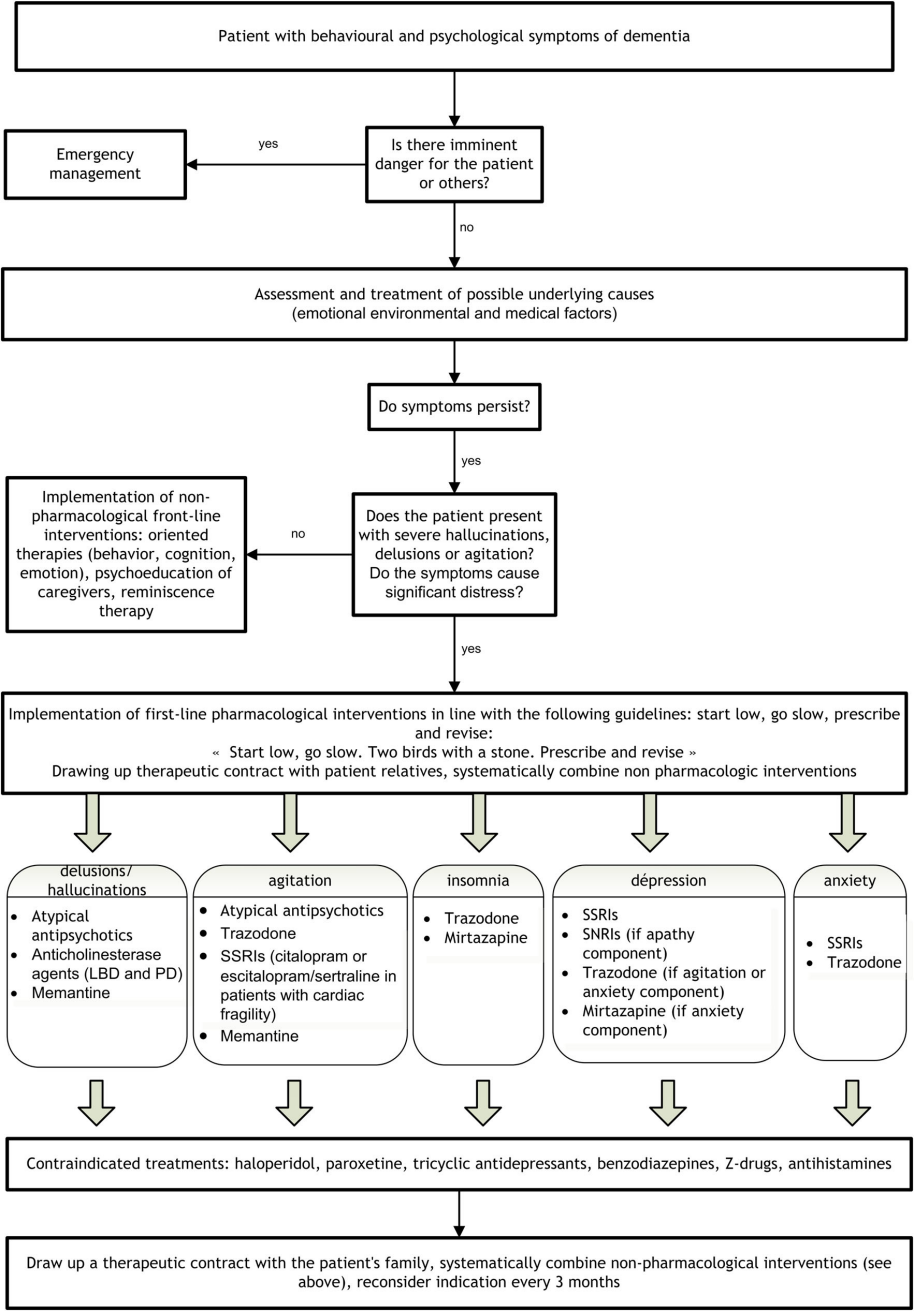
Algorithm for the management of behavioural and psychological symptoms of dementia. LBD, Lewy body dementia; PD, Pakinson's disease; SSRIs, Selective serotonin reuptake inhibitors; SNRIs, serotonin‐norepinephrine reuptake inhibitors.

In this article, we have summarized all the current knowledge required for appropriate management of a patient with BPSD, with the aim of providing clinicians with the theoretical and practical tools they need to gain a detailed understanding of such a complex problem. The choice of treatments to manage psychological and/or behavioural manifestations in a patient with dementia depends on a number of factors which must be carefully considered, such as the origin (primary or secondary) of the BPSD, the severity of the disorders, the impact of psychotropic drugs on neurotransmitter systems, factors inherent in the patient (QT, interactions, risk of bleeding, blood pressure, risk of weight gain, etc.), and the various clinical effects with which psychotropic drugs are associated. With this in mind, we recommend a decision‐making algorithm (Fig. 1) incorporating all the elements developed above.

## DISCLOSURE

The authors have no potential conflicts of interest to disclose.

## Supporting information


**DATA S1.** Causes of BPSD according to the nature of the dementia (AD, FTD, LBD).

**DATA S2.** Summary of recommendations from guidelines developed by scientific and regulatory bodies.

## Data Availability

Data sharing not applicable to this article as no datasets were generated or analysed during the current study.
