# Supplementary material for: The treatment of behavioural and psychological symptoms in dementia: pragmatic recommendations
Source: Psychogeriatrics. 2024 Apr 18;24(4):968–82. doi: 10.1111/psyg.13116 (PMC11578037; doi:10.1111/psyg.13116)
Supplement: Supplementary file 1 — DATA S1. Causes of BPSD according to the nature of the dementia (AD, FTD, LBD). [file PSYG-24-968-s002.docx]

**Supplementary Material 1** Causes of BPSD according to the nature of the dementia (AD, FTD, LBD)

|  | Frontotemporal dementia | Lewy bodies dementia | Alzheimer dementia (early to intermediate stages) |
| --- | --- | --- | --- |
| Delusions/Hallucinations | Atrophy of thalamo-cortico-cerebellar networks | Dysfunction of prefrontal cortex and temporal lobe  Cholinergic hyperactivation of M1 muscarinic receptors | Misinterpretations due to episodic memory impairment  Frontal atrophy Dysregulation of the cortical and subcortical serotonin system |
| Depression | Serotonin dysregulation | Dopaminergic dysregulation | Awareness of cognitive decline  Loss of functional ability  Emotional reaction to progressive cognitive decline |
| Anxiety | Serotonin dysregulation | Pathogenic mechanisms in the cortico-limbic system and corresponding neurotransmitter systems | Awareness of cognitive decline  Loss of self-sufficiency and independence  Secondary symptom of delusions  Disruption of interaction with the environment due to cognitive loss |
| Agitation | Increased activity of dopaminergic neurotransmission | Dysfunction of the parietal lobe and fusiform gyrus | Secondary symptom of depression, anxiety and delusions  Disruption of interaction with the environment due to cognitive loss |
| Sleep Disorders | Orexin dysregulation | Disruption of the pedunculopontine nucleus, the magnocellular reticular formation and the sublasterodorsal nucleus | Infrequent disorders in the early to intermediate stages |
