# Supplementary material for: The treatment of behavioural and psychological symptoms in dementia: pragmatic recommendations
Source: Psychogeriatrics. 2024 Apr 18;24(4):968–82. doi: 10.1111/psyg.13116 (PMC11578037; doi:10.1111/psyg.13116)
Supplement: Supplementary file 2 — DATA S2. Summary of recommendations from guidelines developed by scientific and regulatory bodies. [file PSYG-24-968-s001.docx]

**Supplementary material**

**Summary of recommendations from guidelines developed by scientific and regulatory bodies**

|  | **When should non-pharmacological interventions and pharmacological intervnetions be used in the management of non-cognitive symptoms?** | **How should the treatment of BPSD be monitored?** |
| --- | --- | --- |
| **Organism** | **Recommendation** | **Recommendation** |
| NICE (The National Institute for Health and Care Excellence), United Kingdom^37^ | Before starting non-pharmacological or pharmacological treatment for distress in people living with dementia, conduct a structured assessment to:   - explore possible reasons for their distress and - check for and address clinical or environmental causes (for example pain, delirium or inappropriate care).   As initial and ongoing management, offer psychosocial and environmental interventions to reduce distress in people living with dementia. Only offer antipsychotics for people living with dementia who are either:   - at risk of harming themselves or others or - experiencing agitation, hallucinations or delusions that are causing them severe distress.   Before starting antipsychotics, discuss the benefits and harms with the person and their family members or carers (as appropriate). Consider using a decision aid to support this discussion.  For people living with mild to moderate dementia who have mild to moderate depression and/or anxiety, consider psychological treatments. Do not routinely offer antidepressants to manage mild to moderate depression in people living with mild to moderate dementia, unless they are indicated for a pre-existing severe mental health problem.  Do not routinely offer antidepressants to treat mild to moderate depression in people with mild to moderate dementia, unless they are indicated for a pre-existing severe mental health problem.  Do not offer melatonin to manage insomnia in people living with Alzheimer's disease.  For people living with dementia who have sleep problems, consider a personalised multicomponent sleep management approach that includes sleep hygiene education, exposure to daylight, exercise and personalised activities. | When using antipsychotics:   - use the lowest effective dose and use them for the shortest possible time - reassess the person at least every 6 weeks, to check whether they still need medication.   Stop treatment with antipsychotics:   - if the person is not getting a clear ongoing benefit from taking them and - after discussion with the person taking them and their family members or carers (as appropriate).   Ensure that people living with dementia can continue to access psychosocial and environmental interventions for distress while they are taking antipsychotics and after they have stopped taking them.  For people living with dementia who experience agitation or aggression, offer personalised activities to promote engagement, pleasure and interest.  Do not offer valproate to manage agitation or aggression in people living with dementia, unless it is indicated for another condition. |
| APA (The American Psychiatric Association)^38^ | APA recommends that patients with dementia be assessed for pain and other potentially modiﬁable contributors to symptoms as well as for factors, such as the subtype of dementia, that may inﬂuence choices of treatment.  APA recommends that patients with dementia have a documented comprehensive treatment plan that includes appropriate person-centered nonpharmacological and pharmacological interventions, as indicated.  APA recommends that nonemergency antipsychotic medication should only be used for the treatment of agitation or psychosis in patients with dementia when symptoms are severe, are dangerous, and/or cause signiﬁcant distress to the patient.  APA recommends reviewing the clinical response to nonpharmacological interventions prior to nonemergency use of an antipsychotic medication to treat agitation or psychosis in patients with dementia. | APA recommends that if a risk/beneﬁt assessment favors the use of an antipsychotic for behavioral/psychological symptoms in patients with dementia, treatment should be initiated at a lowdose to be titrated up to the minimum effective dose as tolerated.  APA recommends that if a patient with dementia experiences a clinically signiﬁcant side effect of antipsychotic treatment, the potential risks and beneﬁts of antipsychotic medication should be reviewed by the clinician to determine if tapering and discontinuing of the medication is indicated.  APA recommends that in patients with dementia with agitation or psychosis, if there is no clinically signiﬁcant response after a 4-week trial of an adequate dose of an antipsychotic drug, the medication should be tapered and withdrawn.  APA recommends that in a patient who has shown a positive response to treatment, decision making about possible tapering of antipsychotic medication should be accompanied by a discussion with the patient (if clinically feasible) as well as with the patient’s surrogate decision maker (if relevant) with input from family or others involved with the patient. The aim of such a discussion is to elicit their preferences and concerns and to review the initial goals, observed beneﬁts and side effects of antipsychotic treatment, and potential risks of continued exposure to antipsychotics, as well as past experience with antipsychotic medication trials and tapering attempts.  APA recommends that in patients with dementia who show adequate response of behavioral/ psychological symptoms to treatment with an antipsychotic drug, an attempt to taper and withdraw the drug should be made within 4 months of initiation, unless the patient experienced a recurrence of symptoms with prior attempts at tapering of antipsychotic medication.  APA recommends that in patients with dementia whose antipsychotic medication is being tapered, assessment of symptoms should occur at least monthly during the taper and for at least 4 months after medication discontinuation to identify signs of recurrence and trigger a reassessment of the beneﬁts and risks of antipsychotic treatment. |
| Canadian Medical Association^39^ | As depressive syndromes are frequent in patients with dementia, physicians should consider diagnosing depression when presented with the subacute development (e.g., weeks, rather than months or years) of symptoms characteristic of depression such as behavioural symptoms, weight and sleep changes, sadness, crying, suicidal statements or excessive guilt.  Depressive illness should be treated and when refractory the patient should be referred to a specialist.  Depressive symptoms that are not part of a major affective disorder, severe dysthymia or severe emotional lability, should initially be treated nonpharmacologically.  In patients suffering from disturbing emotional lability or pathological laughing and crying, consider a trial of an antidepressant or mood stabilizer.  Serious behavioural and psychological disturbances are commonly found in people with dementia. Family doctors should ask caregivers about such disturbances and regularly assess their patients. Evaluation to rule out treatable or contributory causes should be done with new onset of agitation, aggression, psychotic behaviour, sleep disturbance or wandering. Environmental (e.g., changes in light or sound stimulation level) and behavioural modifications should be attempted first, often with advice from the Alzheimer Society of Canada and specialists. | If medications are required for the symptomatic control of agitation, aggression or psychotic behaviour, consider low doses of neuroleptic drugs, a serotonin reuptake inhibitor or trazodone.  For sleep disturbances, consider trazodone.  After successful control of symptoms with pharmacotherapy, regularly evaluate the need for continuing treatment and consider withdrawal of medication with close monitoring for emerging symptoms. |
| Best Practice Advocacy Centre New Zealand^40^ | Medicines for BPSD should be only considered once potentially reversible causes have been excluded and non-pharmacological interventions have been trialled; unless there is an immediate risk to the patient or others, or the patient is very severely distressed Always used in combination with non-pharmacological interventions. | Initiate as a trial and not prescribe indefinitely without need; review response to treatment, dose and adverse effects at least every three months  Routinely withdrawn, slowly, after three months of improved symptoms unless symptoms were severe or due to a co-morbid psychiatric disorder, e.g. bipolar disorder or major depression; this is often possible without symptom re-emergence  Re-start at the lowest effective dose, if symptoms return following a withdrawal, and schedule a further trial withdrawal in three to six months  Antipsychotic medicines for the management of BPSD should be initiated as a trial and should not be prescribed indefinitely; treatment should ideally not exceed three months. Initiate at the lowest dose likely to provide therapeutic benefit, e.g. half the adult dose or less, depending on body weight, co-morbidities and concurrent medicine use. Consider timing the dose in relation to the target behaviour, e.g. lunchtime for patients with agitation in the late afternoon. |
| National Clinical Effectiveness Committee (NCEC), Ireland^41^ | Prior to considering any psychotropic medication in a person with dementia, a comprehensive assessment should be performed, by an appropriately trained healthcare professional.  Non-pharmacological interventions should be used initially to treat non-cognitive symptoms in a person with dementia, unless there is severe distress, or an identifiable risk of harm to the person and/or others.  Antipsychotic medication should be used with caution and only in cases where there is aggression, agitation or psychosis that either causes an identifiable risk of harm to the person with dementia and/ or others or causes severe distress to the person.  In people with mild to moderate dementia, and mild to moderate depression and/or anxiety, psychological treatments should be considered. Antidepressants may be considered to treat severe comorbid depressive episodes in people with dementia, or moderate depressive episodes that have not responded to psychological treatment. | Atypical (second generation) antipsychotic medications are associated with fewer extrapyramidal effects and risks than typical (first generation) antipsychotics, and therefore second generation medication should be used if antipsychotic therapy is necessary for the management of non-cognitive symptoms.  If a risk and benefit assessment favours the use of antipsychotic medication, treatment should be initiated at the lowest possible dose and titrated slowly, as tolerated, to the minimum effective dose.  If there is a positive response to treatment with antipsychotic medication, decision making about possible tapering of the medication should occur within 3 months, accompanied by a discussion with the person with dementia and/or their relevant Decision Supporter.  If antipsychotic treatment is being tapered, assessment of symptoms for re-emergence should occur regularly during tapering, and for a period after discontinuation of antipsychotic medication. |
| American Geriatrics Society and American Association for Geriatric Psychiatry^42^ | Recommendations on Depression:  Residents with suicidal ideation, with or without verbalization of a plan to harm themselves, should be considered for immediate referral to a mental health professional for consideration of treatment. (The determination of the need for immediate referral should be based on the particular circumstances, including intent, likelihood of harm to self, and the availability of staff for observation.)  Residents who have depression with psychotic features or who have not responded to 6 or more weeks of treatment should be referred to a mental health professional. (The panel recognizes that access to qualiﬁed mental health professionals may be limited for some facilities. Qualiﬁed primary healthcare providers may be able to perform such services when mental health providers are not available.)  The panel supports the use of nonpharmacological interventions in combination with antidepressant medications for treating major depression.  For residents with minor depression, treatment alternatives include nonpharmacological interventions, antidepressants, and watchful waiting. The choice among them depends upon factors such as severity, previous history, and preferences of the resident, family (if resident desires), or legal representative.  Psychotherapeutic modalities, including group and individual cognitive-behavioral psychotherapy, may be helpful in treating selected residents. Other nonpharmacological interventions supported by the panel include increasing social activities and providing meaningful activities, such as sheltered workshops, volunteering, religious activities, or activities that maintain residents’ past roles.  First-line treatment of major depression should include antidepressant medications  Recommendations on Behavioral Symptoms Associated with Dementia :  Residents who threaten or attempt harm to self or others, with or without inﬂicting actual harm, should be considered for immediate referral to a mental health professional for consideration of treatment. (The determination of need for immediate referral should be based on the particular circumstances, including likelihood of harm to self or others and the availability of staff for observation.)  After associated medical conditions are assessed and treated, the initial treatment of behavioral symptoms should be nonpharmacological when there are no psychotic features and when there is no immediate danger to the resident or others.  Trained professionals or trained nursing home staff should administer appropriate nonpharmacological interventions, which include sensory therapy, activities therapy, modiﬁcation of activities of daily living care to meet individuals’ needs, environmental modiﬁcations, behavioral theory treatments, and social contact interventions.  Appropriate ﬁrst-line pharmacological treatment of residents with severe behavioral symptoms with psychotic features, such as hallucinations and delusions that are causing distress, consists of atypical antipsychotics. | Combination pharmacotherapy for severe behavioral symptoms and psychotic features can be considered after two different trials with two different classes of agents at sufﬁcient doses.  Pharmacological treatments, when used only for dementia-related behavioral symptoms, should be evaluated for tapering or discontinuation not more than 6 months after symptoms are stabilized, followed by attempts at tapering or discontinuation thereafter every 6 months. |
| National health Service (NHS), United Kingdom^43^ | Those who do develop non-cognitive symptoms or behaviours that are severe and distressing should at first be assessed to exclude alternative causes, such as physical health issues (pain/infection), side effects of medication, environmental factors, psychosocial factors, individual biography (e.g. religious beliefs) etc.  Non-pharmacological approaches should always be used as a FIRST LINE in treating behavioural problems associated with dementia.  Pharmacological management is NOT a substitute for non-pharmacological approaches. Non-pharmacological strategies should be continued alongside pharmacological treatment.  Pharmaceutical management strategies should be considered on an individual patient basis. When considering the appropriate strategy any predicted benefits must outweigh any anticipated risks for the individual. Pharmacological strategies should only be considered if the BPSD symptoms cause the individual severe distress and/or there is immediate risk of harm to others. | If, based on an individual risk-benefit analysis, pharmacological management is considered appropriate this should be considered as a therapeutic trial. A review date, at least every 6 weeks, should be set for assessing the benefit and adverse effect of the pharmacological management strategy. If there is no benefit or the patient experiences adverse effects by the review date the medication should be withdrawn.  With all medications always start with a low dose and then slowly titrate up gradually (“START LOW AND GO SLOW”).  Before prescribing antipsychotics, prescribers should:  Target symptoms should be identified and other causes for these symptoms should be ruled out. (patients should be assessed for pain and other potentially modifiable factors to symptoms as well delirium, pain, medical medications, environmental factors, and personal needs as a potential causes of behavioral disturbances)  The choice of antipsychotic should be made following an individual risk/benefit analysis. Including:   - Reviewing patients cerebrovascular risk factors. (Due concern should be given to those with risk factors such as: aged over 80, obesity, diabetes, hypertension, smoking, cardiac arrhythmias); - Previous antipsychotic history; - Adverse effect profile (e.g. movement disorders and Parkinson’s Disease/Dementia with Lewy Bodies).   There should be a full discussion with the patient and/or carers regarding possible benefits/risks of treatment. Cerebrovascular risk factors should be assessed and the possible increased risk of stroke/transient ischaemic attack and possible adverse effects on cognition discussed. (Consider if a decision aid may be useful)  A review date must be set at least every 6 weeks, or sooner if patient is an inpatient, to review target symptoms, adverse effects and cognition. |
| French National Authority for Health (HAS)^44^ | Psychotropic treatment should not be initiated if the symptoms are of somatic or iatrogenic origin.  Psychotropic drugs may be used when appropriate care techniques are not sufficiently effective, particularly when the severity of the behavioural disorders endangers the patient, impairs his or her functioning, or is a threat or a major source of suffering for those around him or her. It is recommended that they be used in synergy with care techniques.  It is not recommended to prescribe psychotropic treatment as a first-line treatment in cases of opposition, shouting or wandering without prior assessment. | Review regular prescriptions and consider continuous or iterative treatment as potentially unnecessary or harmful;  Document the symptomatic targets chosen and the expected impact before initiating treatment;  Choose a product on the basis of the target symptoms and the risk of side-effects;  Favour monotherapy;  Start with small doses, increasing cautiously in stages, and maintain treatment at the best-tolerated effective dose;  Prescribe for the shortest possible duration, with the exception of antidepressants (prescribe in accordance with recommendations for the treatment of major depressive episodes);  Choose the galenic form according to the patient's acceptability. The preferred route of administration should be oral. An injectable form should only be used when urgent treatment of a disorder is required and there is an immediate major risk to safety, when the oral route is not possible and when the parenteral route has been shown to be more effective more quickly;  Reassess signs frequently, as fluctuations are characteristic of behavioural disorders. Assessment at the beginning and at the end of treatment may lead to behavioural problems being misjudged, and often does not guarantee effective treatment;  If possible, change only one treatment at a time, if numerous products are used and found not to be effective;  Teach the patient and family to recognise the positive and undesirable effects of the treatment.. |
| International Psychogeriatric Association^45^ | Consensus outcomes showed a clear preference for an escalating approach to the management of BPSD in AD commencing with the identiﬁcation of underlying causes. For BPSD overall and for agitation, caregiver training, environmental adaptations, person-centered care, and tailored activities were identiﬁed as ﬁrst-line approaches prior to any pharmacologic approaches. If pharmacologic strategies were needed, citalopram and analgesia were prioritized ahead of antipsychotics. In contrast, for psychosis, pharmacologic options, and in particular, risperidone, were prioritized following the assessment of underlying causes. T | no details on monitoring |
| Sube Banerjee’s Report for the Minister of State for Care Services^46^ | Antipsychotics should not be a first-line treatment except in circumstances of extreme risk and harm.  The first line of management should be detailed assessment to identify any treatable cause of the BPSD (eg delirium, pain, depression); this should include taking the history of the problem, having the behaviour described by the carer/team, discussing current and past behaviour with the carer/team.  All treatable causes should be treated with the correct specific treatment (eg antibiotics for infection or antidepressants for depression).  An analysis should be made of whether the behaviour (eg reversal of sleep–wake cycle so that the person with dementia is awake at night) is a problem primarily for the person with dementia, or for their carers (be they paid carers or family carers).  There is a high rate of spontaneous recovery (or placebo effect) in trials, so watchful waiting may be useful in the case of less severe problems since up to half of all cases may be self-limiting.  Benign complementary approaches may be used.  Where intervention is needed, psychological approaches such as structured social interaction should be used in the first instance.  Where medication is considered, the person to whom it is to be given should be as involved as possible in decision-making, although many will lack the capacity to consent. In all cases relatives, particularly the main family carer, and other carers or advocates should be involved in discussions about the use of the medication. They should be given information on the possible positive and negative effects of the medication and be invited to contribute fully to the discussion. Ultimately the decision on whether to prescribe the medication or not will be a ‘best interests’ decision.  Where behaviour is severe and complicated and medication is indicated, then an atypical antipsychotic is to be preferred over a typical one. | Where medication is considered, the person to whom it is to be given should be as involved as possible in decision-making, although many will lack the capacity to consent. In all cases relatives, particularly the main family carer, and other carers or advocates should be involved in discussions about the use of the medication. They should be given information on the possible positive and negative effects of the medication and be invited to contribute fully to the discussion. Ultimately the decision on whether to prescribe the medication or not will be a ‘best interests’ decision.  Where behaviour is severe and complicated and medication is indicated, then an atypical antipsychotic is to be preferred over a typical one.  The medication should be used at the lowest possible effective dose, for the shortest possible time, ideally less than 12 weeks.  Once initiated, the drug’s continuation should be reviewed regularly (at least monthly); at review, reduction or cessation of the medication should be actively considered. |
| Johns Hopkins Memory and Alzheimer’s Treatment Center, and John Hopkins Alzheimer’s Disease Research Center, Baltimore, Maryland^47^ | The panel consensus was that psychotropic drugs should be used only after signiﬁcant efforts have been made to mitigate NPS using behavioral and environmental modiﬁcations and medical interventions if needed, with three exceptions. In each of these cases, use would follow a concern regarding signiﬁcant and imminent risk: major depression with or without suicidal ideation, psychosis causing harm or with great potential of harm, and aggression causing risk to self or others. | The panel reinforced the need for close follow-up to monitor for adverse effects potentially caused by psychotropic medications and that use should be time-limited, because behaviors and symptoms may resolve over time with or without drug treatment. If providers elect to use psychotropics, it is important to remember that there is no Food and Drug Administration approval for their use in the treatment of NPS and that the risk:beneﬁt ratio of medication use must be carefully evaluated. |
| Innovation Center for Neurological Disorders, Xuan Wu Hospital, Capital Medical University, Beijing, China, Department of Neurology, Xuan Wu Hospital, Capital Medical University, Beijing, China^48^ | When the patients with severe agitation and/or aggressiveness that could probably lead to harming themselves or others, antipsychotic medication is needed, but haloperidol (typical antipsychotic) is not recommended as a priority.  When patients with dementia have mild to moderate depression, the use of conventional antidepressants il not recommended (unless there are indications for serious mental illness)  When patients with dementia are accompanied by insomnia, antipsychotics and melatonin should not be recommended.  When patients with dementia also show agitation/aggressive behaviour, relaxation, social interaction, sensory therapy (e.g. music, aromatherapy), structured activities, and behavioural therapy may be adopted.  When patients with dementia also have insomnia, sleep. Hygiene education and personalized multi-component sleep management methods e.g. exposure to daylight, exercise and personalised activities could be adopted | When antipsychotics are considered, the choice of antipsychotics should be based on the risk-benefit ratio.  When administering antipsychotics for 4 to 6 weeks, or tapering the medication for 1-2 weeks, the patients should be regularly evaluated for symptoms.  When patients with dementia have been treated with antipsychotic drugs up to 3 months, or no significant clinical response founded after 4 weeks of therapy, antipsychotic medicine should be gradually reduced and discontinued. |
| Ministry of Health and Social Services; Government of Quebec^49^ | First eliminate the causes of BPSD, listed below, before prescribing medication.  - Eliminate delirium.  - Treat any physical or psychiatric conditions that may be linked to BPSD.  - Stabilise the person's medical condition or optimise their treatment.  - Adjust the patient's medication if pain is being treated sub-optimally.  - Adjust medication to take account of side-effects, withdrawal and drug interactions.  - Stabilise any existing premorbid psychiatric pathology.  - Control, with a view to eliminating, the abuse of certain substances, including the potential abuse of benzodiazepines, opiates and alcohol, and the ensuing effects.  - Meet basic needs.  - Evaluate the approach of staff and loved ones with a view to correcting it, if necessary.  - Identify the psychosocial causes associated with BPSD.  - Eliminate environmental causes.  Intervene first with non-pharmacological approaches to BPSD, mainly when symptoms are mild or moderate and do not present any form of danger to the person or to others.  - Pharmacological approaches are recommended in the following cases.  - When BPSD is severe.  - When they are dangerous.  - In a state of emergency.  - In cases of severe psychological distress.  When pharmacological approaches are indicated, it is recommended that they be used in combination with non-pharmacological approaches.  The symptoms for which medication is not indicated as a first choice or is ineffective are as follows.  - Inappropriate behaviour with regard to elimination of faeces and urine or dressing.  - Repetitive crying (not related to pain or depression).  - Verbal disinhibition.  - Wandering.  - Running away.  - Repetitive movements.  - Orality.  - Resistance to care (hygiene, clothing).  - Accumulation rituals.  If the behaviour is likely to respond to medication and there is no potential non-pharmacological intervention, use the clinical process described in the "Planning and intervention" section of diagram 1 (i.e. include a non-pharmacological intervention in combination with a pharmacological intervention).  Ensure that the person and those around them are sufficiently responsible to ensure that the medication is taken as prescribed. | Try one drug at a time.  Check the person's pharmacological profile to avoid drug interactions.  Remember that age-related changes in metabolism may require the use of smaller doses in the elderly. That said, the elderly are a heterogeneous group, and drug dosing requires an individualised approach for each person.  Increase doses slowly (i.e. q. 1 week, except in emergencies) up to the maximum permitted, adjusting according to the patient's response and tolerance. A complete response may take two to six weeks.  Regularly assess the presence of treatment-related side effects.  Optimise the dose and duration (twelve weeks) of the therapeutic trial according to the patient's tolerance and the effectiveness of the medication, then aim for a stable maintenance dose (i.e. a minimum effective dose) to avoid the continued use of PRNs (Pro re nata or « when required »). NRPs are often associated with side effects and withdrawal or rebound symptoms.  If, after two to four weeks of treatment, the drug is found to be ineffective, consider switching to another drug. In such cases, wean the patient off the drug by gradually reducing the initial treatment and gradually introducing another drug, a method known as "cross-over".  Reassess after three months of behavioural stability.  Attempt a gradual withdrawal of psychotropic drugs after three to six months of behavioural stability. |
| International Psychogeriatric Association^50^ | Antipsychotics should never be used as a first-line approach. Non-pharmacological interventions should be tried first. The benefits should be expected to outweigh the adverse events.  APs should only be prescribed in  (a) symptoms caused by underlying psychotic disorder that causes severe distress to patient/risk to others,  (b) in non-psychotic patients in an extreme and acute situation with risk i.e. severe and harmful physical aggression to oneself or other, severe physical exhaustion, and severe eating/drinking disorders with a risk of malnourishment or dehydration.  The behavior is not caused by another somatic disorder (such as pain, infection, hunger, constipation) or  psychiatric disorder (anxiety/depression).  Only antipsychotics with proven evidence should be prescribed. | Start low, go slow.  Investigation of underlying syndromes, neurological, psychiatric, environmental (interaction) factors.  Assessment of medical state and risk (cardiovascular and subtype of dementia (Lewy Body/Parkinson) and  symptoms (motor symptoms, cardiac arrhythmias, orthostatic hypotension, urine retention).  ECG should be carried out in patients with history of cardiovascular diseases, cardiac arrhythmia, and combination of medication that prolong QT-interval.  Care and treatment plan should draw expertise form multidisciplinary team/with regular consultation.  Family caregiver should be informed and consulted throughout treatment and discontinuation.  Improvement and lack of improvement should be included as a clinical criterion for modifying care and treatment plan.  Discontinuation should be a standard principle as part of a withdrawal plan.  If APs are prescribed for sedative purposes, drug should be withdrawn when situation has calmed down.  Discontinuation through tapering rather than immediate discontinuation unless Malign Neuroleptic Syndrome,  cardiovascular complication, infections, severe side effect at low dose.  Long-term antipsychotic treatment is only acceptable in patients with   - long history or high severity of psychotics/concurrent schizophrenia, - at least two unsuccessful discontinuation attempts + psychosocial interventions has been shown not to be effective + alternative medication is not available/has been shown ineffective/expected to cause severe adverse events.   Restarting can be acceptable – under supervision of a specialist – in extreme situation in case of   - recurrence of severe symptoms after withdrawal resulting in risk/distress that had previously improved with AP treatment, - recurrence of severe symptoms after withdrawal if withdrawal was before completing a 12-week course, - a distinct separate new episode. |
